# Not your usual drug-induced liver injury: Dolutegravir-associated granulomatous hepatitis

**DOI:** 10.4102/sajhivmed.v27i1.1817

**Published:** 2026-06-30

**Authors:** Muhammed Vally, Muhammad Ayob, Adam Mahomed, Kairoonisha Mahomed, Washington Mudini, Vikash Lala

**Affiliations:** 1 Division of Medical Gastroenterology, Department of Internal Medicine, Faculty of Health Sciences, University of the Witwatersrand, Johannesburg, South Africa; 2 Division of Medical Gastroenterology, Department of Internal Medicine, Wits University Donald Gordon Medical Centre, Johannesburg, South Africa; 3 Private Practice, Johannesburg, South Africa; 4 Department of Anatomical Pathology, Faculty of Health Sciences, University of the Witwatersrand, Johannesburg, South Africa; 5 Department of Anatomical Pathology, Wits University Donald Gordon Medical Centre, Johannesburg, South Africa

**Keywords:** dolutegravir, drug-induced liver injury, granulomatous hepatitis, hepatotoxicity, liver biopsy, antiretroviral therapy, cholangitis, HIV

## Abstract

Dolutegravir is an integrase inhibitor in first-line antiretroviral therapy in South Africa. We describe a patient who developed severe drug-induced liver injury on dolutegravir-containing antiretroviral treatment, in whom liver biopsy demonstrated subacute hepatic necrosis, non-necrotising granulomatous inflammation, and bile duct injury: an uncommon histological pattern in dolutegravir-associated hepatotoxicity.

**What this study adds:** This case highlights the histopathological spectrum of dolutegravir-induced liver injury, documenting a rare granulomatous–cholangitic pattern. We aim to raise awareness of dolutegravir hepatotoxicity and caution against misclassification as infectious or autoimmune hepatitis. The case also underscores the diagnostic value of liver biopsy in atypical antiretroviral-associated liver injury.

## Introduction

Dolutegravir (DTG), a second-generation integrase inhibitor, is widely used in antiretroviral therapy because of its high efficacy, strong genetic barrier to resistance, and favourable safety profile.^1^ Although generally well tolerated, DTG has been implicated in rare cases of drug-induced liver injury (DILI), which can result in acute liver failure and, in severe cases, liver transplantation.^1,2^

The mechanisms underlying DTG-associated hepatotoxicity are incompletely understood, and may involve mitochondrial dysfunction, oxidative stress, and immune-mediated injury.^2^ Histologically, reported cases most commonly demonstrate cholestatic or mixed patterns of liver injury, with limited biopsy-based descriptions of severe necrotic patterns.^3^ We report a rare and severe presentation of DTG-associated liver injury with unique histopathological features.

## Case presentation

A 39-year-old woman living with HIV presented with a 7-day history of progressive jaundice associated with pruritus and intermittent retrosternal discomfort. She reported social alcohol consumption limited to weekends, totalling less than three units per week. Her past medical history was unremarkable, with no previous history of liver disease. She had been diagnosed 6 months previously with HIV and was initiated on a fixed dose combination of tenofovir-lamivudine-dolutegravir (TLD), with a CD4 count of 350 cells/μL and no current or previous opportunistic infections. A detailed medication and toxin exposure history was obtained, and there was no prior exposure to anti-tuberculosis therapy. She denied the use of over-the-counter (OTC) medications, traditional or herbal remedies, and weight-loss supplements. There was no reported recent exposure to industrial or environmental toxins. She was not receiving co-trimoxazole prophylaxis, isoniazid preventive therapy, or any other hepatotoxic medications.

On initial clinical assessment, she was severely jaundiced but haemodynamically stable, with no features of chronic liver disease or hepatic encephalopathy. Abdominal examination was unremarkable, with no hepatomegaly, ascites, or focal tenderness. An abdominal ultrasound demonstrated a liver of normal contour and morphology, with no focal hepatic lesions and no intrahepatic or extrahepatic bile duct dilatation. The gallbladder was distended and contained multiple gallstones, the largest measuring 18 mm × 7 mm, with gallbladder wall thickening of 4 mm and no pericholecystic fluid. The common bile duct measured 3 mm, with no choledocholithiasis identified.

Liver function testing demonstrated severe hepatic dysfunction with a marked cholestatic–hepatocellular pattern, including marked hyperbilirubinaemia, severe transaminitis, and an elevated International Normalised Ratio (INR) of 1.47 at presentation. Hepatitis A, B, and C serology was negative, and other baseline investigations were unremarkable (Table 1 to Table 4). A magnetic resonance cholangiopancreatography (MRCP) was performed and showed no dilatation of the intrahepatic or extrahepatic biliary tree and no obstructing calculi. The gallbladder wall appeared thickened and oedematous, with multiple small calculi. Furthermore, no focal liver lesions, contour abnormalities, or hepatomegaly were identified.

**TABLE 1:** Serial haematological, biochemical, and ancillary laboratory investigations at initial visit.

Laboratory investigation	Outcome 15 September 2025
**FBC**	
WCC (×10^9^/L)	5.63
Hb (g/dL)	12.9
MCV (fL)	82.8
Platelets (×10^9^/L)	356.0
**U/E**	
Na (mmol/L)	140.0
K (mmol/L)	4.2
Cl (mmol/L)	102.0
HCO_3_ (mmol/L)	24.0
Urea (mmol/L)	5.0
Creatinine (μmol/L)	65.0
**Inflammatory markers**	
CRP (mg/L)	18.9
**Additional investigations**	
*Treponema pallidum* total (serum)	Negative

FBC, full blood count; WCC, white cell count; Hb, haemoglobin; MCV, mean corpuscular volume; U/E, urea and electrolytes; Na, sodium; K, potassium; Cl, chloride; HCO_3_, bicarbonate; CRP, C-reactive protein.

**TABLE 2:** Serial laboratory investigations demonstrating the evolution of liver biochemistry.

Laboratory investigation	Outcome						
	15 September 2025	17 September 2025	25 September 2025	02 October 2025	08 October 2025	14 October 2025	23 January 2026
Total protein (g/L)	49	50	67	72	72	69	78
Albumin (g/L)	24	24	34	34	34	37	42
ALT (IU/L)	1233	1178	270	365	140	87	54
AST (IU/L)	1514	1423	181	392	137	60	43
ALP (IU/L)	229	245	235	302	227	265	147
GGT (IU/L)	279	294	471	648	575	796	422
Total bilirubin (μmol/L)	296	286	136	136	73	53	8
Conjugated bilirubin (μmol/L)	222	220	101	89	30	39	1.1
INR	1.47	1.34	1.17	1.07	1.12	-	1.10

ALT, alanine aminotransferase; AST, aspartate aminotransferase; ALP, alkaline phosphatase; GGT, gamma-glutamyl transferase; INR, international normalised ratio.

**TABLE 3:** Immunoglobulin profile and autoimmune serological workup.

Laboratory investigation	Outcome (15 September 2025)
**Immunoglobulins**	
IgG (g/L)	20.8
IgA (g/L)	2.56
IgM (g/L)	1.3
IgG4 (g/L)	0.31
**Autoimmune workup**	
ANA	Positive (1:80)
dsDNA	Negative
Smooth muscle antibody	Negative
AMA-M2	Negative
M2-3E (BPO)	Negative
Sp-100	Negative
PML	Negative
gp210	Negative
LKM-1	Negative
LC-1	Negative
SLA/LP	Negative
Ro-52	Negative
C-ANCA (PR3)	Negative
P-ANCA (MPO)	Negative
C-ANCA IFA titre	Negative
P-ANCA IFA titre	Negative

IgG, immunoglobulin G; IgA, immunoglobulin A; IgM, immunoglobulin M; IgG4, immunoglobulin G subclass 4; ANA, antinuclear antibody; dsDNA, double-stranded DNA antibody; AMA-M2, antimitochondrial antibody M2; BPO, branched-chain 2-oxoacid dehydrogenase complex; PML, promyelocytic leukaemia protein; gp210, glycoprotein 210 antibody; LKM-1, liver-kidney microsomal antibody type 1; LC-1, liver cytosol antibody type 1; SLA/LP, soluble liver antigen/liver-pancreas antibody; Ro-52, Ro52 antibody; C-ANCA, cytoplasmic antineutrophil cytoplasmic antibody; PR3, proteinase 3; P-ANCA, perinuclear antineutrophil cytoplasmic antibody; MPO, myeloperoxidase; IFA, indirect immunofluorescence assay.

**TABLE 4:** Viral serology and HIV investigations.

Laboratory investigation	Outcome (15 September 2025)
**Viral studies**	
Hepatitis A IgM	Negative
Hepatitis B core Ab	Negative
Hepatitis B surface antigen	Negative
HBsAg value	0.40 S/CO
Hepatitis B surface Ab	Immune
HBsAb value	406.09 mIU/mL
Hepatitis C antibody	Negative
Smooth muscle antibody	Negative
HIV-1/2 (4th Gen)	Reactive
HIV-1/2 (4th Gen) confirmatory	Reactive
HIV-1 RNA	Not detected

IgM, immunoglobulin M; Ab, antibody; HBsAg, hepatitis B surface antigen; HBsAb, hepatitis B surface antibody; S/CO, signal-to-cut-off ratio; mIU/mL, milli-international units per millilitre; Gen, generation.

Following withdrawal of TLD, the patient demonstrated substantial biochemical improvement with downtrending bilirubin and transaminase levels with progressive normalisation of the INR during admission. However, there was a transient secondary rise in transaminases and cholestatic enzymes, particularly gamma-glutamyl transferase (GGT) and alkaline phosphatase (ALP), despite an overall improving biochemical trajectory (Table 2). This prompted initiation of corticosteroid therapy, namely oral prednisone, at a dose of 20 mg in the context of worsening transaminase levels, elevated immunoglobulin G, and a weakly positive autoimmune screen, raising concern for an immune-mediated process. Corticosteroids were subsequently tapered. As corticosteroid therapy had already been initiated prior to liver biopsy, the degree of inflammatory activity on histology may have been partially modified by treatment, a consideration that was taken into account when interpreting the biopsy findings. In view of the persistent and unexplained liver injury despite initial medical management, a decision was made to proceed with liver biopsy.

**FIGURE 1:**
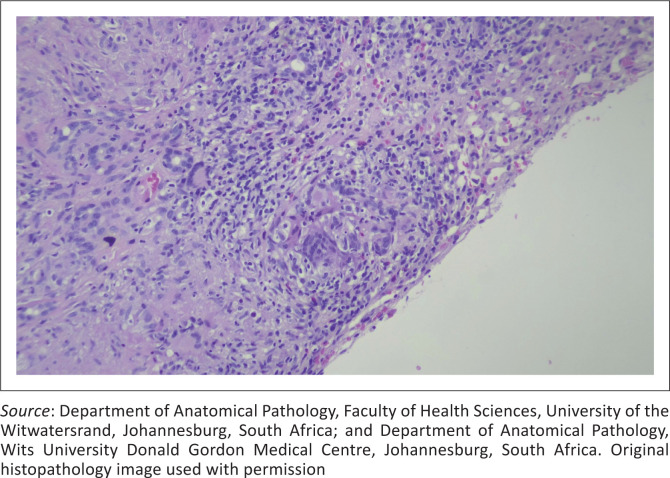
Liver biopsy demonstrating subacute hepatic necrosis with non-necrotising granulomas, consistent with granulomatous–cholangitic drug-induced liver injury.

Liver core biopsy demonstrated preserved lobular architecture with extensive subacute hepatic necrosis and reticulin collapse (Figure 1). Portal tracts were expanded by a mixed inflammatory infiltrate comprising lymphocytes, plasma cells, neutrophils, histiocytes, eosinophils, and numerous non-necrotising granulomas composed of epithelioid histiocytes and multinucleated giant cells, occasionally surrounding small bile ducts. Bile duct epithelial injury and ductular proliferation were present, with mild interface activity. Notably, there was no prominent plasma cell clustering or significant plasma cell participation in the interface activity. Special stains were negative for acid-fast bacilli and fungi. In addition, mycobacterial tuberculosis polymerase chain reaction performed on liver tissue was negative, and liver tissue culture for *Mycobacterium tuberculosis* showed no growth. Overall, the findings were most consistent with a granulomatous–cholangitic pattern of DILI, likely associated with DTG.

She was followed up at 1 month and at 4 months, during which she demonstrated progressive clinical and biochemical improvement. She had resolution of jaundice and pruritus with a significant improvement, though not complete normalisation, of her liver function tests (Table 2). In the context of suspected DTG-associated DILI, the patient was switched to a non-DTG-containing antiretroviral regimen consisting of lopinavir/ritonavir (LPV/r) and rilpivirine (RPV). HIV care remained uninterrupted following this change, with stable CD4 counts and sustained virological suppression.

## Case discussion

DTG-associated hepatotoxicity is uncommon, and is most frequently described as mild, transient liver enzyme elevation, typically manifesting as cholestatic or mixed hepatocellular–cholestatic injury.^2^ While liver enzyme abnormalities have been reported in patients receiving DTG-based regimens, clinically significant DILI remains rare, and progression to severe hepatic failure has been documented only in isolated case reports.^3^ Importantly, despite extensive global use of DTG, biopsy-proven cases of severe DTG-associated DILI are rare, with most published literature relying on clinical and biochemical criteria rather than histological confirmation.^2,4^ Consequently, the histopathological spectrum of DTG-induced liver injury remains poorly characterised.

The most striking feature of this case was a granulomatous–cholangitic pattern of liver injury, characterised by extensive subacute zonal and bridging necrosis, numerous portal-based non-necrotising granulomas, and bile duct epithelial injury with ductular proliferation. While severe necrosis may be seen in immune-mediated or toxic injury, granulomatous inflammation with a cholangitic component is not a recognised dominant pattern in reported cases of DTG-associated hepatotoxicity, underscoring the atypical nature of this presentation.

Notably, despite marked biochemical improvement following DTG withdrawal, our patient demonstrated a transient secondary rise in transaminases and cholestatic enzymes prior to eventual recovery. This biphasic pattern has been described in cholangitic forms of DILI, and may reflect delayed resolution of bile duct epithelial injury despite improvement in the initial hepatocellular insult.^5^ Cholestatic DILI is recognised to resolve more slowly than hepatocellular injury, and may demonstrate fluctuating biochemical abnormalities during recovery because of ongoing ductular inflammation and cholangiocyte injury.^5,6^ Additionally, the granulomatous–cholangitic histological pattern in our case supports a possible immune-mediated mechanism, whereby hepatic inflammation may transiently persist, even after withdrawal of the offending agent.^5,6^

Accordingly, granulomatous hepatitis in individuals living with HIV presents a broad differential diagnosis, including infectious causes such as tuberculosis and fungal disease, immune-mediated conditions such as sarcoidosis or autoimmune hepatitis, and DILI.^7^ In this case, infectious aetiologies were considered unlikely given the absence of necrotising granulomas, negative special stains for acid-fast bacilli and fungi, and lack of ancillary clinical or radiological features suggestive of disseminated tuberculosis or other infections.

Autoimmune hepatitis (AIH) was therefore considered in view of the patient’s gender, antinuclear antibody (ANA) positivity (1:80), and elevated IgG level (22.4 g/L). Using the 2008 Simplified International Autoimmune Hepatitis Group (IAIHG) criteria,^8^ she accrued 2 points for ANA positivity, 2 points for elevated IgG (> 1.1 × upper limit of normal), and 2 points for exclusion of viral hepatitis, with histology considered at most ‘compatible’ (1 point), yielding a cumulative score of 6–7, which approaches the threshold for probable AIH (≥ 6). However, the biopsy lacked typical features of AIH and instead demonstrated granulomatous inflammation, which is distinctly uncommon in this condition. Furthermore, recent International AIH Pathology Group (IAIHPG) consensus recommendations emphasise injury topography and portal-based plasma cell-rich inflammation as key diagnostic features,^9^ which were absent in this case.

Although the simplified score approached the threshold for probable AIH, the absence of typical histological features, and a relevant drug exposure history, are incompatible with classification as likely AIH under established diagnostic frameworks. Additionally, elevated IgG and ANA positivity may reflect non-specific immune activation in the context of HIV infection rather than true autoimmune hepatitis.^9^ Taken together, the overall clinicopathological picture was most suggestive of a DTG-associated DILI, although the atypical granulomatous–cholangitic pattern and overlapping immune-mediated features warrant cautious interpretation. Using the Roussel Uclaf Causality Assessment Method (RUCAM),^10^ the patient achieved a score of 6, consistent with probable DILI. DTG was considered the most likely causative agent in the absence of alternative toxin exposure or concomitant hepatotoxic medications.

## Conclusion

In this case, liver biopsy was essential after non-invasive investigations failed to establish a unifying diagnosis, providing definitive evidence of a granulomatous–cholangitic pattern with subacute necrosis, and allowing exclusion of infectious and primary autoimmune causes. This case highlights that DTG-associated hepatotoxicity may present with granulomatous and cholangitic features, broadening the diagnostic framework for unexplained liver injury in individuals living with HIV, and underscoring the value of liver biopsy in complex presentations.
